# Players’ On-Court Movements and Contextual Variables in Badminton World Championship

**DOI:** 10.3389/fpsyg.2020.01567

**Published:** 2020-07-10

**Authors:** Raúl Valldecabres, Claudio A. Casal, João Guilherme Cren Chiminazzo, Ana María de Benito

**Affiliations:** ^1^Doctorate School, Valencia Catholic University San Vicente Mártir (UCV), Valencia, Spain; ^2^Physical Activity and Sports Science Faculty, Valencia Catholic University San Vicente Mártir (UCV), Valencia, Spain; ^3^Centro Universitário de Jaguariúna (Unifaj), Jaguariúna, Brazil

**Keywords:** observational methodology, match analysis, motor behavior, situational variables, badminton

## Abstract

This study aimed to analyze the elite badminton players’ on-court movements related to contextual variables (game, round, and match status). A total of 18 matches of the Jakarta 2015 World Championship (1,273 points and 5,710 play actions) were examined by univariate and bivariate analyses. Significant differences were found when comparing the players’ on-court movements related to game, round, and match status (*p* < 0.05). All movements were executed more frequently in game 2, with the exception of diagonal large backward left (DLBL), diagonal short backward left (DSBL), diagonal short backward right (DSBR), and longitudinal short backward (LSB). The results obtained related to the round showed that longitudinal large backward (LLB) was the most frequent footwork in R1/16 and R1/2, diagonal short forward left (DSFL) was the most frequent one for R1/4, and transversal short right (TSR) was the most used movement for the final round. According to match status, no movement (NM) was the most common situation before hitting the shuttlecock at any moment during the match. This study shows how contextual variables modulate the elite players’ on-court movements. This information could be valuable for coaches and players, allowing them to better understand the players’ behavior in a competition, which could be used to design more specific training tasks and prepare match strategies in order to improve the players’ performance in competitions.

## Introduction

Badminton is one of the most popular racket sport in the world (Phomsoupha and Laffaye, 2015). Practice increased after inclusion in the 1992 Olympic Games and after the scoring system changed, making it a faster and more exciting sport (Chen et al., 2011). It requires athletes to be in good physical shape to perform fast and sudden movements such as jumps, landings, and changes of direction (Shariff and Ramlan, 2009; Kuntze et al., 2010; Phomsoupha and Laffaye, 2014) to hit the shuttlecock from different positions (Phomsoupha and Laffaye, 2015) and perform different kinds of shot (Cabello, 2000). To determine the game demands, researchers need to analyze precisely the different match movements and movement patterns (Hughes et al., 2009). Going into detail about knowledge of performance factors is crucial for methodological approaches and for a more general understanding of sport sciences (Drust, 2010).

Badminton is a highly explosive and intense sport (Kuntze et al., 2010; Reilly et al., 2013), and players require very fast footwork to reach the shuttlecock and back to the center of the court (Phomsoupha and Laffaye, 2014). However, researchers have showed little attention to the performance factors related to the players’ on-court movements. In other racket sports, scientific knowledge of the players’ footwork is more extensive. For example, in table tennis, Pradas et al. (2012) indicated that table tennis players perform technical gestures at maximum speed together with brief and swift traveling steps to constantly change direction, as it occurs in badminton. Malagoli Lanzoni et al. (2007) defined the different types of steps used by table tennis players and their frequency. Malagoli Lanzoni and Lobietti (2009) confirmed that the step most often used was the one step, even for players form different levels (Malagoli Lanzoni et al., 2013a). Malagoli Lanzoni et al. (2013b) found out that one step was the step most often used by males (31.9%), while stroke without step was the one most often used by females (43.6%). In tennis, some studies have analyzed the direction of the players’ movement (Weber et al., 2007), indicating that a tennis player performs 72% of his movement along the baseline, 17% forward, and 8% backward. Hughes and Meyers (2005) analyzed the movement of tennis players and reported that 67% of all movement was initiated with a split step, which is typically timed to coincide closely with the opponent’s ball contact.

In badminton, as far as we know, only three recent studies about footwork were found. Abdullahi and Coetzee (2017) determined the relationships between singles match strokes and foot movements in male badminton players who participated in the African Badminton Championship, indicating that, on average, the foot movements per match were chasse step (174.6), shuffle (161.7), split-step (61.6), half lunge (52.20), forward lunge (46.1), and scissors kick (38.3). Valldecabres et al. (2017) analyzed the gender differences in the players’ behaviors during the final singles matches of the 2015 Badminton World Championship, focusing on time events, shots, and court movements. They indicated that hitting the shuttlecock without any previous displacement predominates over the rest of the foot movements for both genders. In addition, this study showed that more than 50% of the successful court movements correspond to diagonal. Similarly, it has been shown that, 20% of the time, the players hit the shuttlecock without making any previous movement, which, in turn, is the least successful movement pattern in both finals. Previously, Kuntze et al. (2010), through a video-based pilot study, stated that almost 15% of all the players’ displacements during competitive singles matches were lunges, so they were the most frequent movements in badminton.

Besides that, when the players’ performance is evaluated, researchers should take into account that the competition context is a key factor that can modify it. Several works have analyzed the match based on the game and the competition level in badminton. Recently, Primo et al. (2019) analyzed the interactive effects about the success of the challenge according to the contextual variable identifying that the success of a challenge is affected by who and when the request is made so that the players could improve their strategic plans that involve selecting the most appropriate moment to request the hawk-eye. Chiminazzo et al. (2018) identified that the play-off phase showed higher total time, rest time, points played, and shots per rally when compared with the group stage. The frequency of serve, net, smash, and total shots were also significantly higher in the play-offs. Torres-Luque et al. (2019) found out game differences regarding timing factor in single and double matches from play-off and group phase. Gómez et al. (2019) analyzed timing and technical performance differences between elite men and women’s badminton players and observed gender differences during games 2 and 3 as the match progressed. Abián-Vicén et al. (2013) analyzed 20 singles matches from the Beijing 2008 Olympic Games and did not find timing variable differences between games 1 and 2.

No previous badminton work that analyzes the badminton game performance based on match status variable (draw, lose, and win) has been found, but it has been explored in other sports such as football. According to Casal et al. (2017), the match status influences the players and the teams’ behaviors. These authors analyzed the effects of a match status on corner kick performance indicators in 95 matches played during the final stages of the 2012 UEFA European Championships and the 2010 FIFA World Cup. The results showed that, when a corner kick is taken during the last 30 min of the match, teams that are losing would place six or more attackers in the shooting area, while teams that are drawing would place two to five attackers in this area. In the same situation, the teams that are drawing would place one to two defenders at the goalposts, while the winning teams would place none. For that reason, we hypothesized that similar findings could be found in badminton where players can modulate their behavior depending on their match situation.

To the best of our knowledge, there are no previous studies that analyze the interaction of contextual variables (game, round, and match status) on the players’ on-court movements. For that reason, the purpose of this study was to analyze the elite badminton players’ on-court movements related to contextual variables (game, round, and match status). This information could be used to design more specific and quality training tasks. Furthermore, knowing how contextual categories determine the players’ footwork would be valuable for coaches and players, allowing them to prepare match strategies related to game, round, or opponents’ play level in order to improve their performance in a competition.

## Materials and Methods

### Design and Sample

A systematic observational methodology, with a non-participant observer and in a natural context, was applied since it was considered as the most suitable for its characteristics of natural context, spontaneity, and perceptiveness (Anguera, 1979; Blanco and Anguera, 2003). This work is designed to match the IV quadrant: nomothetic observational design (plurality of units), intersessional following (more than one session throughout the time), and multidimensional (simultaneous and concurrent different answer levels are considered in the observational tool) according to Anguera et al. (2001).

A sample of 18 men’s single matches from the 2015 Jakarta Badminton World Championship was randomly selected and analyzed (1,273 points and 5,710 play actions), with 10 from round 1/64, three from round 1/16, two from round 1/4, two from round 1/2, and one from the final round. Neither round 1/32 nor round 1/8 matches were selected because of the method used to randomize the sample^1^. These players were the best in the world at the time of the analysis.

All videos were taken from the official website of the International Badminton Federation^2^. According to the Belmont Report (1978), the use of public images for research purposes does not require informed consent.

### On-Court Movements and Contextual Variables

**FIGURE 1:**
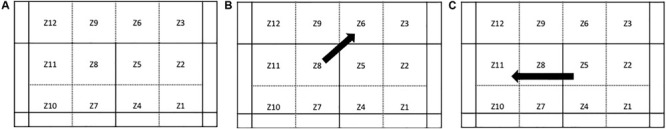
**(A)** Badminton court divided into 12 identical zones. **(B)** Example of a diagonal short forward right movement. **(C)** Example of a transversal large left movement.

The variable “court movement” is defined as the movement trajectory executed by the observed player, taking into account the starting zone and the ending zone. The badminton court was previously divided into 12 identical dimension zones or quadrants (Z1, …, Z12) (Figure 1). The starting zone refers to the quadrant where the observed player is when the opponent hits the shuttlecock, and the ending zone refers to the quadrant where the observed player hits the shuttle back to the opponent’s court.

The players’ on-court movements are classified according to the Badminton Observational Tool previously validated by Valldecabres et al. (2019). They are defined taking as reference the plane in which it is executed – longitudinal (L), transversal (T), and diagonal (D); the distance traveled – short (S), large (L), and no movement (NM); and the direction of the movement, taking as a reference the starting position with respect to the net – left (L), right (R), forward (F), and backward (B). The blend of all the criteria mentioned above results in the next code list: DLBL – diagonal large backward left, DLBR – diagonal large backward right, DLFL – diagonal large forward left, DLFR – diagonal large forward right, DSBL – diagonal short backward left, DSBR – diagonal short backward right, DSFL – diagonal short forward left, DSFR – diagonal short forward right, LLB – longitudinal large backward, LLF – longitudinal large forward, LSB – longitudinal short backward, LSF – longitudinal short forward, NM – no movement, TLL – transversal large left, TLR – transversal large right, TSL – transversal short left, and TSR – transversal short right. Figure 1 shows the court zones and DSFR and TLL movements as examples.

The on-court movement’s comparative analysis has been carried out with the serve actions removed because they are executed from a static position as established by the game rules.

Also, as a contextual variable, match status was noted as *win* (when the player is ahead on score), *lose* (the player is behind in the score), and *draw* (when the game is a tie). The other two contextual variables are *game* and *round.* A previous study has shown different demands according to championship stage, so it should be interesting to see if the on-court movement patterns change during the championship (Chiminazzo et al., 2018; Torres-Luque et al., 2019). In this study, only the first and the second games of each match were considered. The third game of three games was discarded to keep the analysis in concordance with the data being compared to.

### Procedure

The matches were recorded from TV emitted images and were registered and analyzed post-event by three experienced badminton coaches by using the previously validated tool of Valldecabres et al. (2019). LINCE software program (Gabin et al., 2012) was employed for visualizing the recorded matches and registering the qualitative data (game, round, and match status). KINOVEA v.0.8.27 was used to draw the 12 court zones and register the on-court players’ footwork on the 12-zone stencil (see Figure 1), and IBM SPSS Statistics v.23 (SPSS Inc., Chicago, IL, United States) was used for statistical analysis.

Prior to data collection, the observers were trained following the protocols of Losada and Manolov (2015) during 10 sessions using the consensual agreement method among observers as described by Anguera (1990) so that recording was only done when agreement was produced. Inter- and intra-observers’ reliability for match analysis was assessed using Cohen’s Kappa criterion (Cohen, 1960). Intra-observer reliability was completed by the observers, coding five random matches selected from the data sample. Following a 5-week period, to avoid any possible negative learning effects, the matches were recorded and the two data games were compared. Inter-observer reliability was performed by coding of one random match by two observers. Based on the reference criteria proposed by Fleiss et al. (2003), the intra-observer and the inter-observer agreement in the present study can be considered as “almost perfect” (Table 1).

**TABLE 1:** Inter- and intra-observers’ Cohen’s Kappa reliability results.

**Category**	**DLBL**	**DLBR**	**DLFL**	**DLFR**	**DSBL**	**DSBR**	**DSFL**	**DSFR**	**LLB**
Intra-observer agreement	0.976	0.97	0.972	0.978	0.962	0.979	0.963	0.97	0.967
Inter-observer agreement	0.974	0.978	0.979	0.977	0.971	0.972	0.965	0.972	0.980

**Category**	**LLF**	**LSB**	**LSF**	**NM**	**TLL**	**TLR**	**TSL**	**TSR**	***K*_*total*_**

Intra-observer agreement	0.963	0.962	0.964	0.969	0.968	0.977	0.977	0.976	0.971
Inter-observer agreement	0.979	0.971	0.971	0.969	0.974	0.974	0.974	0.973	0.974

### Statistical Analysis

Two different analyses were carried out. First, a univariate analysis was executed to show absolute and relative frequencies and the mean and standard deviation (SD) of the players’ on-court movements related to each contextual variable observed (game, round, and match status). This analysis allows describing the players’ on-court movements referring to match contextual variables. Furthermore, the players’ on-court movements were analyzed separately in trying to determine if the players’ behaviors were modulated by the contextual variables observed and if those relations were statistically significant. The following associations were tested using one-way chi-square (χ^2^) test to determine the association between different situational variables (independent variables) and the dependent variable (players’ on-court footwork): (1) players’ on-court movements within each game, (2) player’s on-court movements within each competition round, and (3) players’ on-court movements in relation to match status. Statistical significance was defined as a *p*-value < 0.05.

## Results

Table 2 shows the descriptive analysis of the players’ on-court movements per game. All movements were executed more frequently in game 2, with the exception of DLFL, DSBL, DSBR, and LSB. DSBL (53.4%) was the commonest one during game 1, while TLL (60.4%) showed higher values on game 2. Furthermore, the players’ on-court movements were associated with game (χ^2^ = 0.075, *p* = 0.027).

**TABLE 2:** Players’ on-court movements per game.

**Category**	**Game 1**		**Game 2**		**Match Mean ± SD**
	**Mean ± SD**	**Frequency (%)**	**Mean ± SD**	**Frequency (%)**	
DLBL	9.76.6	44.8	11.98.7	55.2	10.87.7
DLBR	7.23.2	46.4	8.35.3	53.6	7.84.3
DLFL	6.93.6	50.8	6.73.8	49.2	6.83.7
DLFR	9.86.1	47.1	11.06.9	52.9	10.46.5
DSBL	10.95.8	53.4	9.56.3	46.6	10.26.0
DSBR	6.34.0	52.1	5.83.0	47.9	6.13.5
DSFL	10.54.6	46.9	11.94.4	53.1	11.24.5
DSFR	12.86.0	46.3	14.98.6	53.7	13.97.3
LLB	0.70.9	48.0	0.71.2	52.0	0.71.1
LLF	0.91.3	42.1	1.21.4	57.9	1.11.3
LSB	3.12.0	52.9	2.71.9	47.1	2.91.9
LSF	10.45.6	40.3	15.47.0	59.7	12.96.7
NM	20.413.0	49.9	20.58.4	50.1	20.510.8
TLL	3.12.0	39.6	4.73.6	60.4	3.93.0
TLR	3.92.1	45.2	4.73.1	54.8	4.32.6
TSL	8.33.0	47.2	9.35.5	52.8	8.84.4
TSR	6.95.1	42.8	9.27.2	57.2	8.16.3
Diagonal	74.429.8	56.3	71.934.1	53.9	73.131.7
Longitudinal	15.16.6	11.4	19.19.4	13.5	16.78,2
Transverse	22.17.8	16.8	25.117.1	18.8	23.713.4
NM	20.413.0	15.5	18.58.0	13.8	19.410.6
Large	42.317.8	32.0	44.224.4	33.2	43.321.3
Short	69.321.8	52.5	70.935.2	53.0	10.129.3

*SD, standard deviation; DLBL, diagonal large backward left; DLBR, diagonal large backward right; DLFL, diagonal large forward left; DLFR, diagonal large forward right; DSBL, diagonal short backward left; DSBR, diagonal short backward right; DSFL, diagonal short forward left; DSFR, diagonal short forward Right; LLB, longitudinal large backward; LLF, longitudinal large forward; LSB, longitudinal short backward; LSF, longitudinal short forward; NM, no movement; TLL, transversal large left; TLR, transversal large right; TSL, transversal short left; TSR, transversal short right.*

When analyzing the players’ movements related to the direction traveled, it could be seen that the on-court footwork follows a similar pattern in both games, with diagonal court movements as the most frequently used and longitudinal movement as the least frequently used. In addition, significant differences between games have been found (χ^2^ = 0.047, *p* = 0.011). Diagonal and NM showed higher values in game 1 than in game 2; meanwhile, longitudinal and transverse were more often used in game 2. Analyzing the distance of gathered on-court movements, short court movements were the commonest ones and NM were the least frequently used in both games. However, these results were not statistically significant (χ^2^ = 0.025, *p* = 0.206).

Table 3 shows the analysis of the players’ on-court movements per round. It could be observed that the highest values for all kinds of on-court movement were found in the final round, except DLBR that was more frequent in R1/2 (7.2%), DSFL that was frequently used in R1/4 (7.6%), and LLB (13.3%) and NM (6.8%) that were more frequent in R1/6. Significant differences between the players’ on-court footwork related to that of the championship round have been found (χ^2^ = 0.081, *p* ≤ 0.001).

**TABLE 3:** Players’ on-court movements per round.

**Category**	**R1/64**		**R1/16**		**R1/4**		**R1/2**		**Final**
	**Mean ± SD**	**%**	**Mean ± SD**	**%**	**Mean ± SD**	**%**	**Mean ± SD**	**%**	**%**
DLBL	17.68.7	4.5	23.38.1	6.3	15.012.2	5.8	25.015.6	6.5	12.1
DLBR	13.35.5	4.8	16.72.1	6.0	12.37.2	6.6	20.00.0	7.2	7.1
DLFL	12.04.5	4.9	17.73.8	7.2	7.76.5	4.7	13.57.8	5.5	9.3
DLFR	17.59.1	4.7	26.010.0	7.0	9.03.5	3.6	26.08.5	7.0	11.2
DSBL	18.79.6	5.1	19.74.0	5.4	16.017.4	6.6	20.05.7	5.5	9.0
DSBR	10.73.5	4.9	13.72.1	6.2	6.04.4	4.1	15.53.5	7.1	10.0
DSFL	19.16.6	4.7	28.74.5	7.1	20.32.5	7.6	19.09.9	4.7	6.7
DSFR	25.812.0	5.2	30.77.6	6.1	17.37.5	5.2	32.08.5	6.4	6.6
LLB	1.11.1	4.4	3.31.5	13.3	0.00.0	0	2.02.8	8.0	0
LLF	1.71.6	4.5	3.03.5	7.9	1.01.0	4.0	3.02.8	7.9	7.9
LSB	5.51.5	5.3	5.503.6	4.8	3.32.5	4.8	5.53.5	5.3	12.5
LSF	24.110.0	5.2	32.010.0	6.9	19.35.5	6.2	17.53.5	3.8	7.7
NM	42.516.6	5.8	50.37.4	6.8	21.319.4	4.4	32.04.2	4.4	4.5
TLL	6.03.0	4.3	10.31.5	7.4	5.35.9	5.8	7.52.1	5.4	12.2
TLR	7.93.1	5.1	9.02.0	5.8	5.05.2	4.9	8.04.2	5.2	11.6
TSL	17.27.5	5.4	16.31.5	5.2	13.08.0	6.2	16.56.4	5.2	7.3
TSR	13.87.0	4.8	15.76.1	5.4	10.05.3	5.2	16.05.7	5.5	14.8
Diagonal	134.741.4	4.9	176.335.0	6.4	103.757.1	5.6	171.043.8	6.2	8.9
Longitudinal	32.410.3	5.1	43.312.9	6.8	23.77.5	5.6	28.07.1	4.4	8.2
Transverse	44.914.8	5.0	51.36.4	6.4	33.323.3	5.4	48.07.1	5.6	8.6
NM	42.516.6	5.8	50.37.4	6.8	21.319.4	4.4	32.04.2	4.4	4.5
Large	76.323.2	4.7	109.318.1	6.6	55.338.9	5.1	105.022.6	6.4	10.3
Short	136.949.1	5.1	161.722.4	6.1	105.343.3	6.0	142.035.4	5.4	8.6

*SD, standard deviation; DLBL, diagonal large backward left; DLBR, diagonal large backward right; DLFL, diagonal large forward left; DLFR, diagonal large forward right; DSBL, diagonal short backward left; DSBR, diagonal short backward right; DSFL, diagonal short forward left; DSFR, diagonal short forward right; LLB, longitudinal large backward; LLF, longitudinal large forward; LSB, longitudinal short backward; LSF, longitudinal short forward; NM, no movement; TLL, transversal large left; TLR, transversal large right; TSL, transversal short left; TSR, transversal short right.*

Focusing on the plane and the distance of gathered on-court movements, it could be observed that in the first round (1/64) of the championship, NM was the commonest situation when hitting the shuttlecock. In the middle rounds, the players regularly performed longitudinal court movements, while in R1/2 and in the final round, the mostly used footwork was that of diagonal movements. Statistically significant differences were found for all variables (χ^2^ = 0.057, *p* < 0.001). Regarding the analysis of distance covered by gathered on-court movements, significant differences between rounds could be identified (χ^2^ = 0.057, *p* < 0.001). Specifically, in rounds R1/64 and R1/16, NM is where the players often hit the shuttle. In R1/4, short footwork was the commonest displacement, while in R1/2 and in the final round, large movements were the most frequent player movements.

Analyzing the players’ on-court movements per match status (Table 4), it can be observed that DLBL, LLB, LLF, LFS, and TSR were the most frequently performed movements when the player is down on the scoreboard, while DLBR, NM, TLL, and TSL were the most frequently executed movements when the player is winning the score. There was a significant association between the players’ on-court movements and the match status (χ^2^ = 0.070, *p* < 0.000).

**TABLE 4:** Players’ on-court movements per match status.

**Category**	**Draw**		**Lost**		**Win**	
	**Mean ± SD**	**%**	**Mean ± SD**	**%**	**Mean ± SD**	**%**
DLBL	2.93.0	0.6	8.88.5	0.5	9.86.3	0.5
DLBR	1.61.8	11.0	6.05.3	12.7	8.04.7	13.8
DLFL	1.61.9	1.5	5.54.3	3.4	6.64.2	1.8
DLFR	3.63.3	4.8	9.06.5	4.1	8.24.5	3.3
DSBL	2.82.0	2.9	7.45.6	2.2	10.26.1	2.6
DSBR	1.51.4	13.6	5.63.8	13.5	5.13.4	12.8
DSFL	3.72.7	6.7	8.15.1	5.1	10.66.0	5.6
DSFR	3.74.0	8.8	11.46.8	7.4	12.66.5	7.7
LLB	0.20.4	0.3	0.81.2	0.7	0.40.8	0.3
LLF	0.20.4	1.0	1.01.2	1.0	0.91.1	0.7
LSB	0.91.4	2.0	2.21.8	1.9	2.71.5	1.9
LSF	3.82.8	8.1	12.26.1	10.3	9.97.5	7.4
NM	5.24.0	23.5	17.210.1	22.0	18.612.6	27.8
TLL	1.31.6	0.0	3.23.1	0.1	3.23.2	0.2
TLR	1.41.4	4.5	3.43.5	3.9	3.72.4	3.7
TSL	2.42.3	4.5	5.94.6	4.4	9.23.7	4.6
TSR	1.72.3	6.1	6.68.2	6.8	7.85.9	5.5
Diagonal	21.415.7	55.40	61.937.5	54.10	70.933.4	55.71
Longitudinal	5.13.9	13.09	16.28.0	14.13	13.98.3	10.95
Transverse	6.95.3	17.99	19.217.9	16.76	23.911.8	18.76
NM	5.24.0	13.53	17.210.1	15.01	18.612.6	14.57
Large	12.89.6	33.23	37.727.6	32.98	40.820.3	53.36
Short	20.614.9	53.23	59.534.4	52.01	67.930.6	32.07

*SD, standard deviation; DLBL, diagonal large backward left; DLBR, diagonal large backward right; DLFL, diagonal large forward left; DLFR, diagonal large forward right; DSBL, diagonal short backward left; DSBR, diagonal short backward right; DSFL, diagonal short forward left; DSFR, diagonal short forward right; LLB, longitudinal large backward; LLF, longitudinal large forward; LSB, longitudinal short backward; LSF, longitudinal short forward; NM, no movement; TLL, transversal large left; TLR, transversal large right; TSL, transversal short left; TSR, transversal short right.*

Table 4 also shows that whatever the match status was, diagonal movements were the most frequently used, while longitudinal movements were the least performed by the badminton players, and these differences were statistically significant (χ^2^ = 0.036, *p* = 0.048). Diagonal and transverse movements were more frequent when the player is ahead on the scoreboard, while longitudinal and NM movements were more executed when the player is behind on the scoreboard. On the other hand, a comparative analysis of the gathered on-court movements related to distance covered by players per match status showed different patterns, although statistically significant differences have not been found (χ^2^ = 0.012, *p* = 0.816). Large footwork was more frequent when the players were winning, NM movements were commonly used when they were losing the score, and short ones were the most frequently executed when the players were drawing.

## Discussion

Data referring to the players’ on-court movement categories per game provide statistically significant results (χ^2^ = 0.075, *p* = 0.027). The most frequently executed movements mentioned above are commonly used in the defensive players’ strategy in terms of shots (Cabello, 2000) or when it is not possible to get a good hit of the shuttlecock but with enough time to send it to a place far away from the opponent. The onset of game 2 on a fatigued state would be the reason why the players are not able to perform good footwork along the court (Girard and Millet, 2009), combined with weak positional play, which result in losing the court position or executing forced hits (Lees, 2003). Moreover, the players get a reduction on force and powerful capacity on a fatigued state, so the fatigued players could tend to perform “natural hitting,” which could be defined as the most natural joint movement instead of trying to make a more precise and technical one (Enoka, 2008).

Comparing plane gathered on-court movements per game shows statistically different patterns (χ^2^ = 0.047, *p* = 0.011), which could be due to the increase of net and lob shots in game 2 because of the onset of fatigue. It produced a lower diagonal footwork by not allowing the ability to repeatedly perform sprints and strokes (Girard et al., 2008) where the players approach the net, so in game 2, the players’ need to do fast lineal (longitudinal and transverse footwork) movements to hit the shuttlecock. These results are close to those obtained by Valldecabres et al. (2017). However, there are some differences that could be explained because these authors analyzed all shots, without discarding serves. Moreover, their sample corresponds to both genders when it is known that the players’ behavior differs between men and women (Abián-Vicén et al., 2013). On the other hand, NM shows lower values for game 2, which could be due to the onset of the players’ fatigue or the technical modifications affected by fatigue, which would imply the execution of very slow on-court movements or not as fast as needed along the court for them to recover the defensive position (Girard and Millet, 2009).

The comparative analysis of the players’ on-court movements per round shows different behavioral patterns when compared across championship rounds (χ^2^ = 0.081, *p* = 0.001). In particular, in Table 4, it could be seen as different on-court frequency varies between rounds and, in each one of them, different on-court footwork patterns were performed. In R1/64, the most frequent footwork was NM, perhaps because the athletes did not seek an aggressive game since they were identifying the strategies of the opponent.

In terms of results according to footwork plane, diagonal movements were the most performed whatever the round was (*p* = 0.001), which coincides with the results of Valldecabres et al. (2017). Perhaps this result was obtained due to the fact that they are players of high technical level and, that said, they always try to anticipate the shuttle, always moving diagonally to hit it and thus reducing the opponent’s response time. In addition, the analysis of distance covered by gathered on-court movements per round showed statistically significant differences (χ^2^ = 0.057, *p* = 0.001), indicating that NM was the lowest value percentage for the final round. That could be explained by the players’ technical and tactical skills, which forced the opponent to carry out a greater number of court movements.

Analyzing the players’ on-court movements per match status, it could be seen that the players’ footwork pattern is influenced by this variable (χ^2^ = 0.070, *p* = 0.000). At all moments during the match, NM was the most common situation before hitting the shuttlecock. That seems to confirm our hypothesis mentioned above: the new badminton playing style implies hitting the shuttlecock back to the opponent’s body instead of doing it to the further opponent’s corner as per the proposal of Cabello (2000). This strategy would reduce the chance of hitting the shuttle back. Other on-court footwork commonly used were DLBR and DSBR because sending the shuttlecock far from the opponent is part of badminton’s internal locus, while TLL was the least frequently used movement because it is an easy return for the opponent, shifting from offensive to defensive phase. In addition, it has been checked that most of the footwork were performed when the scoreboard was in draw, except DLBL, LLB, LLF, LFS, and TSR that were predominantly performed when losing the score or DLBR, NM, TLL, and TSL that were commonly used when winning in the scoreboard.

Data obtained for plane gathered on-court movement according to match status showed significant differences (χ_2_ = 0.036, *p* = 0.048), which was similar to the results of Valldecabres et al. (2017). It was verified that diagonal footwork was the most frequent at any scoreboard result. It is considered as a natural way of playing badminton because the players need to widen the court to recover the offensive strategy, independent of the scoreboard. This may be due, as Cabello (2000) states, to the badminton internal locus, which consists of sending the opponent to the far side of where he is waiting for the next shot, making diagonal movements regardless of the scoreboard. In addition, it has to be noticed that longitudinal and NM footwork were performed mainly when the player was down on the score, perhaps due to a not technically good shot which prevents one from performing skilled maneuvers. On the contrary, when the players were up on the scoreboard, the players performed higher transversal and diagonal footwork.

A comparative analysis of the distance gathered on-court movements depending on match status showed a greater value for large footwork when the player was leading the match. It could be assumed that the winning players are capable of making a greater number of long court movements, indicating that they are fitter to reach that court area which the opponent has sent the shuttlecock to. The obtained results confirmed the initial hypothesis and the situational variables analyzed to modulate the players’ on-court behavior.

One of the main limitations of this study is that only the 2015 World Championship was studied, so it would be necessary to broaden the sample by analyzing a larger number of matches corresponding to the National Leagues in order to extrapolate the findings. In addition, game 3 could add valuable information about the players’ behavior under a fatigued state and should be analyzed in future works. The differences shown by the statistical analyses give information about the players’ strategy changes all through the championship; however, it is still not clear where these differences come from. A deeper analysis on this line should be performed in future lines to know about the variances. Finally, it is important to mention that the interaction of the on-court players’ footwork results shown previously could not be compared with that of other studies due to the absence of related information.

## Conclusion

The game behavior differences of high-level players (best players in the world at the moment when this research was carried out) focusing on game, round, and match status have been studied. In general, all movements were executed more frequently in game 2, while different on-court movements were observed depending on the round, and according to match status, NM was the most common situation before hitting the shuttlecock at any moment during the match. The main conclusion taken from the results is that the contextual variables analyzed (game, round, and match status) have significant correlations with the on-court movements executed by elite badminton players during the championship matches, which demonstrates that the players may modify their behavior due to fatigue or a change in their strategy. With this information, the players could improve their endurance to get to the end of the match with less fatigue, which could result in a better championship score. The information shown above could provide a deep understanding of the players’ game behavior. Therefore, coaches and players could use it as a source document in order to design more specific training tasks and match strategies that will enhance the players’ performance in competitions.

## Data Availability Statement

The datasets generated for this study are available on request to the corresponding author.

## Author Contributions

RV registered the data, reviewed the literature, and wrote the manuscript. CC developed the project, wrote the manuscript, and was responsible for performing analysis and methods and writing the statistical analysis sections. AB developed the project, wrote, translated, and drafted the manuscript. JC drafted the manuscript. All authors contributed to the article and approved the submitted version.

## Conflict of Interest

The authors declare that the research was conducted in the absence of any commercial or financial relationships that could be construed as a potential conflict of interest.

## Footnotes

1 www.random.org

2 www.bwfbadminton.com
